# Age-period-cohort analysis of hepatitis A incidence rates in Korea from 2002 to 2012

**DOI:** 10.4178/epih.e2016040

**Published:** 2016-09-30

**Authors:** Joo Yeon Seo, Sungyong Choi, BoYoul Choi, Moran Ki

**Affiliations:** 1 Department of Preventive Medicine & Institute for Health and Society, Hanyang University College of Medicine, Seoul, Korea; 2 Gyeonggi Infectious Disease Control Center, Seongnam, Korea; 3 Department of Cancer Control and Policy, Graduate School of Cancer Science and Policy, National Cancer Center, Goyang, Korea

**Keywords:** Epidemiology, Hepatitis A, Incidence

## Abstract

**OBJECTIVES:**

This study aimed to evaluate the epidemiology of hepatitis A in Korea from 2002 to 2012 using age-period-cohort analyses.

**METHODS:**

We used claims data from the Korean National Health Insurance Corporation for the entire population. Census data from 2010 were used as the standard population. The incidence of hepatitis A was assumed to have a Poisson distribution, and the models and effects were evaluated using the intrinsic estimator method, the likelihood ratio, and the Akaike information criterion.

**RESULTS:**

The incidence of hepatitis A gradually increased until 2007 (from 17.55 to 35.72 per 100,000 population) and peaked in 2009 (177.47 per 100,000 population). The highest incidence was observed among 27-29-year-old individuals when we omitted data from 2005 to 2007. From 2005 to 2007, the peak incidence was observed among 24-26-year-old individuals, followed by 27-29-year-olds. The best model fits were observed when the age-period-cohort variables were all considered at the same time for males, females, and the whole population.

**CONCLUSIONS:**

The incidence of hepatitis A exhibited significant age-period-cohort effects; its incidence peaked in 2009 and was especially high among Koreans 20-39 years of age. These epidemiological patterns may help predict when high incidence rates of hepatitis A may occur in developing countries during their socioeconomic development.

## INTRODUCTION

Hepatitis A virus (HAV) is mainly transmitted through the fecal-oral route, although transmission can also involve eating contaminated food or person-to-person transmission [1,2]. Lifelong immunity is often acquired after HAV infection [3], and hepatitis A during childhood is generally asymptomatic or causes flu-like symptoms. However, it is also associated with symptoms that range from nausea and vomiting to fulminant hepatitis and death among adults [1,2,4,5]. Unfortunately, hepatitis A is one of the most common infectious diseases in the world [6], and its incidence varies according to socioeconomic development and public sanitation. The endemicity of hepatitis A is generally high or intermediate in developing countries [6-8], and low in developed countries [8]. Furthermore, epidemiological shifts can occur within a country or birth cohort group, based on socioeconomic developments and public sanitation improvements [7]. Moreover, these characteristics are pronounced in countries that have experienced high levels of socioeconomic growth, such as South Korea (hereafter Korea) [7,8]. Therefore, it is important to evaluate the incidence patterns of hepatitis A according to age, period, and birth cohort, in order to understand such epidemiological shifts and to develop suitable public policy initiatives. This study aimed to determine the epidemiological characteristics of hepatitis A in Korea from 2002 to 2012, based on age-period-cohort (APC) analyses.

## MATERIALS AND METHODS

### Ethical statement

The retrospective design of this study was reviewed and approved by the institutional review board of Hanyang University (HYI-15-024-2).

### Data source

We used claims data from the Korean National Health Insurance Corporation. These data included sex, age, the patient’s address, disease type, date of diagnosis, and medical history. Cases of hepatitis A were identified using the International Classification of Diseases, 10th revision codes B15, B15.0, and B15.9. In cases of repeated treatment for the same diagnosis, the first claim was used for the analyses. The annual mid-year populations were provided by Statistics Korea.

### Statistical analysis

Population and housing census data from 2010 were used as the standard population for calculating the age-standardized incidence of hepatitis A. APC analyses were used to identify the age, period, and cohort effects of hepatitis A. The category of age was divided into 3-year groups, with the exception of a ≥81-year-old group based on the low incidence of hepatitis A in that group. The time periods were defined as 2002-2004, 2005-2007, 2008-2010, and 2011-2012. Birth cohorts were defined on the basis of 3-year cohorts from 1922 to 2012, and individuals who were born before 1921 were included in a single cohort. The incidence of hepatitis A was assumed to have a Poisson distribution, and the APC effects were measured using the intrinsic estimator (IE) method [9]. The optimal model was selected based on the likelihood ratio and the Akaike information criterion. All analyses were performed using SAS version 9.4 (SAS Institute Inc., Cary, NC, USA).

## RESULTS

Hepatitis A incidence gradually increased starting from 2002, and peaked at 83,414 individuals in 2009 (Table 1). The patterns of incidence according to sex were similar. The overall incidence according to age from 2002 to 2012 was highest in individuals 30-39 years old (102,065 individuals), followed by 20-29-year-olds (93,175 individuals).

Table 2 presents the age-standardized hepatitis A incidence per 100,000 population according to sex and birth year from 2002 to 2012. In 2009, the incidence of hepatitis A in the overall population and males peaked in the 1978-1980 cohort (29-31 years old; 459.99 and 529.46 per 100,000 population, respectively). The incidence in the 1981-1983 cohort of females (26-28 years old) peaked at 408.43 per 100,000 population. The 1975-1986 cohorts (25-35 years old) generally exhibited the highest incidence rates, although some differences were found between males and females in the peak incidence rates according to cohort and age at diagnosis.

**Figure 1.**
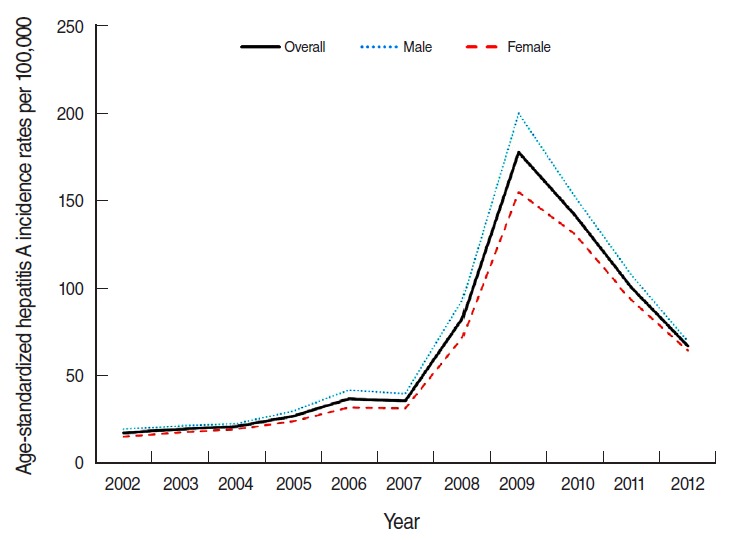
Age-standardized hepatitis A incidence rates per 100,000 population according to sex in Korea.

**Figure 2.**
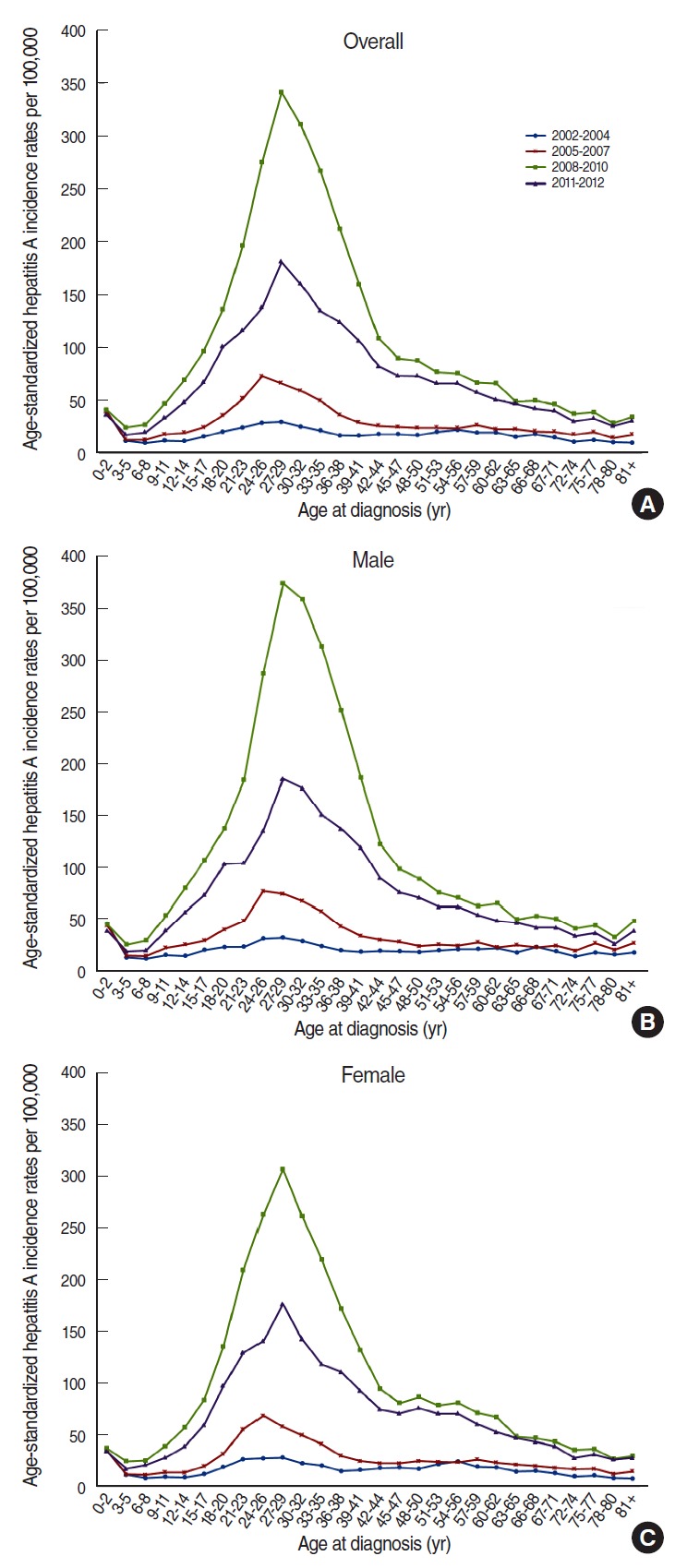
Trends in hepatitis A incidence rates per 100,000 population according to age and period in Korea. (A) Overall population, (B) male, and (C) female.

Figure 1 shows the incidence of hepatitis A during from 2002 to 2012; it gradually increased from 2002 to 2007 (from 17.55 to 35.72 per 100,000 population, respectively) and peaked in 2009 (177.47 per 100,000 population). The incidence subsequently decreased to 67.16 per 100,000 population in 2012. When we omitted the data from 2005 to 2007, the highest incidence was observed among 27-29-year-old individuals. From 2005 to 2007, the highest incidence was observed for 24-26-year-old individuals, followed by individuals who were 27-29 years old. The incidence rates for males and females were similar, although they were slightly higher for men (Figure 2).

**Figure 3.**
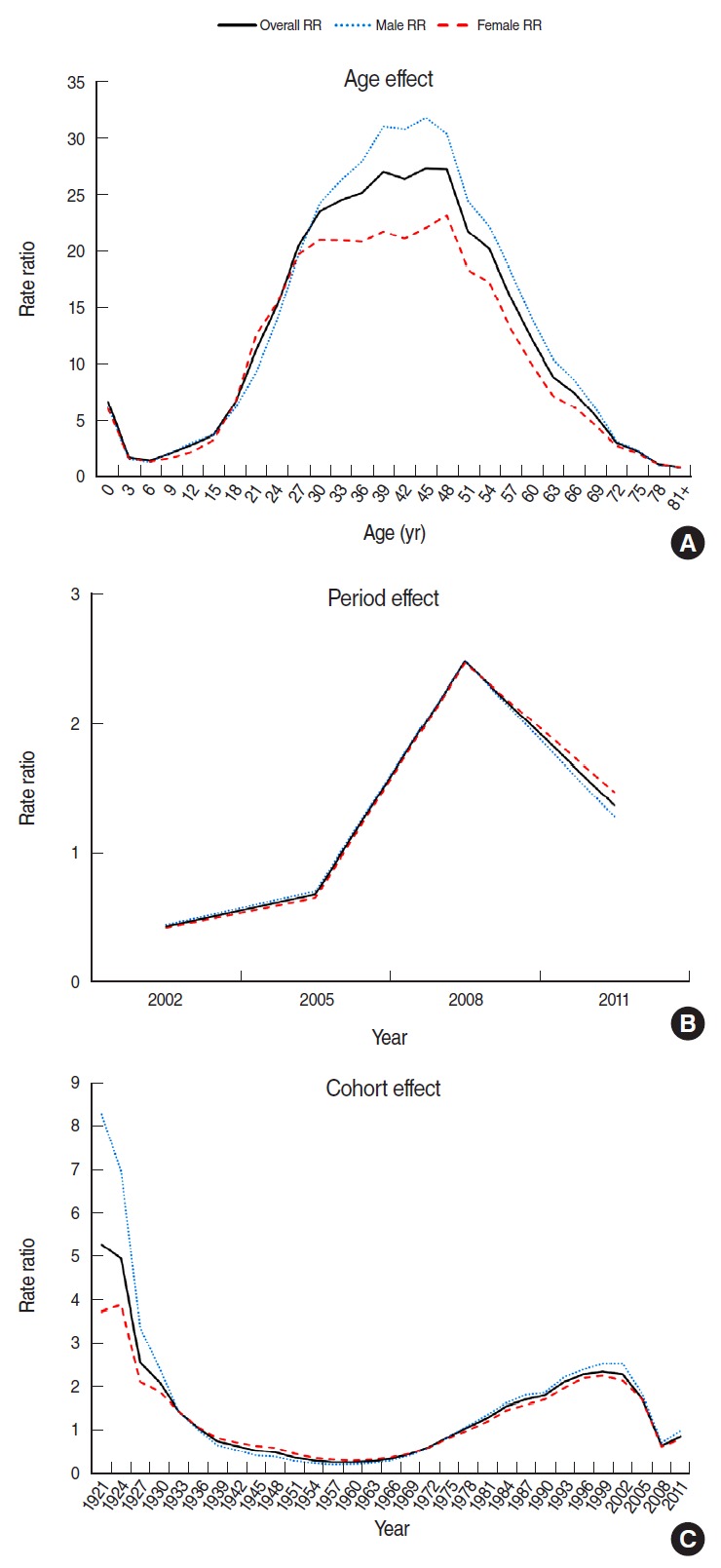
Age-period-cohort analysis of hepatitis A incidence in Korea. (A) Age effect, (B) period effect, and (C) cohort effect.

The APC analyses revealed that the best model fits for males, females, and the overall populations were observed when all APC variables were considered at the same time (Table 3). Figure 3 shows the APC effect using IE models. After correcting for the period and cohort effects, a high incidence was observed for 0-2-year-old individuals, and the incidences subsequently decreased until the age of 6-8 years. The incidence rates gradually increased starting with the age of 9-11 years, peaked in the late 30s, and subsequently decreased with age (Figure 3A). After correcting for age and cohort effects, the incidence of hepatitis A was found to have increased from 2002-2004 through 2008-2010, and then decreased beginning in 2011. Similar patterns were observed for males and females (Figure 3B). After correcting for age and period effects, the incidence of hepatitis A was highest in the pre-1921 birth cohort, decreased until the 1957-1959 cohort, and subsequently increased until the 1999-2001 cohort (Figure 3C).

## DISCUSSION

We performed APC analyses of hepatitis A in Korea from 2002 to 2012 using nationally representative data. These analyses revealed that the full APC model provided a better fit than the age, period, age-period, age-cohort, and period-cohort models. Therefore, the APC model was best suited for our analysis.

Korea is a good example of the transition from a poor country to a developing country and subsequently to a developed country; these transitions influenced public health issues, such as hepatitis A epidemiology. For example, the per capita Korean gross domestic product remained at <200 USD between the Korean War (1950-1953) and 1968, although it has subsequently increased to approximately 27,000 USD in 2015. This economic development and concomitant improvements in public sanitation have altered the epidemiology of hepatitis A in Korea, and the endemicity of hepatitis A has changed from high to intermediate [10]. In this context, various studies have evaluated the incidence, seroprevalence, risk factors, and vaccination status for hepatitis A [3,4,11-15], and have reported high incidence rates among individuals who are 20-39 years old. Nevertheless, it is likely that recent changes in age cohorts, vaccination status, lifestyle, and overseas travel have influenced the incidence of hepatitis A.

The present study revealed that hepatitis A did not exhibit an increasing age effect, as the age-specific incidence was highest among young adults who were 20-39 years old. This result is consistent with the reported age-specific seroprevalence rate in Korea [11,12], as the high seroprevalence in individuals <10 years of age is related to vaccination that was introduced in 1997 [4], and the high seroprevalence in individuals >40 years of age is related to childhood infections that occurred before Korea’s rapid socioeconomic development and public sanitation improvements [11-13]. Therefore, hepatitis A occurred most frequently in the 20-39-year-old group, who exhibited a low seroprevalence rate. However, the incidence rapidly increased after the age of 18-20 years, which is when most individuals start college or develop an active social life. These adults were likely infected by HAV during overseas travel to endemic areas, after consuming shellfish, or at catered social events [4,16-19]. In addition, the incidence rates of hepatitis A were highest in 2009 for the 1978-1980 male birth cohort and the 1981-1983 female birth cohort. Although a difference of three years was found between males and females, the patterns confirm that the highest incidence was observed among individuals who were in their mid-20s.

Analyzing hepatitis A incidence rates per 100,000 population, a gradually increasing pattern was seen from 2002 to 2007, followed by a sudden increase in 2008 and 2009. The cause of this spike in incidence is thought to be cases of sporadic occurrences in many regions, as observed in 2008 in the Gwangju region by Seo et al. [10] and an increase in laboratory diagnoses due to the rapid growth of awareness of hepatitis A.

We observed a high incidence of HAV among 0-2-year-old children; although hepatitis A is generally mild and self-limiting among children, some cases involving complications have been reported [20,21]. In addition, some cases have required liver transplantation [22,23], which indicates that symptomatic and/or severe hepatitis A is possible among children. Furthermore, awareness among parents and pediatricians (based on the hepatitis A explosion during the late 2000s) has likely increased the number of children who have undergone diagnostic testing [24]. Thus, the increased incidence of hepatitis A among very young children may be related to the increased awareness and testing of patients with non-specific symptoms in that age group.

A period effect was observed from 2008 to 2010, and the incidence of hepatitis A subsequently decreased after that point. This effect was likely related to a vaccination program that was introduced in Korea in 1997 [4]. Furthermore, the Korea Centers for Disease Control and Prevention started a national childhood vaccination program in May 2015 [3], which will likely help reduce the future incidence of hepatitis A.

Variable birth cohort effects were observed, with high incidence rates observed in the pre-1930 cohorts and the 1980-1999 cohorts (20-39 years old at onset). Furthermore, the incidence of hepatitis A for the post-1978 cohorts increased continuously, although rapid decreases were observed in the post-2000 cohorts. Several studies of immunoglobulin G anti-HAV seropositivity have shown U-patterns with specific susceptible age groups. The birth years of such groups reported in previous studies have ranged approximately from 1970 to 2000, making those findings consistent with those of our study [11-13]. Various factors have likely influenced these effects. For example, these dramatic changes are closely related to the economic development of Korea, which was relatively weak until 1970, until which point Korea was characterized by poor public health, sanitation, and individual hygiene [10]. Thus, cohorts from this period likely had anti-HAV antibodies because they were exposed to HAV during their childhood [12]. In contrast, subsequent socioeconomic and sanitation improvements likely delayed HAV exposure in the post-1980 cohorts [11,13]. Nevertheless, a risk of exposure is still present, as Korean public sanitation has not yet improved to the level of advanced countries that have low HAV endemicity [10].

The changes in the Korean endemicity of hepatitis A are likely a good example for developing countries, as hepatitis A is exhibiting a transition from high endemicity to intermediate endemicity in several Asian countries (e.g., China, India, and Malaysia) and Middle Eastern countries (e.g., Iran, Iraq, the United Arab Emirates, and Lebanon) [25-27]. Furthermore, hepatitis A in the Middle East may spread rapidly, given the poor hygiene, unclean water, and dense populations in Syrian refugee camps [27]. Moreover, hepatitis A vaccination is not currently sufficient in the Middle East, despite hepatitis A vaccination being included in several national immunization programs (e.g., in Bahrain, Cyprus, Iraq, Israel, Saudi Arabia, and Qatar) [27]. Therefore, since Korea has undergone rapid economic development, the patterns of change for hepatitis A in Korea may help predict future patterns in these countries.

This study has several limitations. First, we were not able to determine the incidence of asymptomatic infections [10], as many children have subclinical symptoms of hepatitis A, and it is possible that we underestimated the incidence of hepatitis A in this age group. Second, we only evaluated data from an 11-year period, which is relatively short. However, hepatitis A is typically an acute disease that is cured within six weeks [2], and this study period was likely sufficient for evaluating the incidence and changing patterns of hepatitis A. Third, the health insurance data using our study were secondary data based on diagnostic codes used for insurance claims. Therefore, our findings may have been slightly different from those that would have been obtained using a gold-standard test for the disease based on laboratory identification of the immunoglobulin M anti-HAV antibody. Despite these limitations, our findings provide a good model of the changing epidemiological patterns of hepatitis A, which are based on Korean socioeconomic growth patterns that are currently reflected in Middle Eastern and Asian countries. Our results may help predict high incidence patterns that could occur in developing countries. If these countries are not properly prepared, outbreaks of hepatitis A may pose a significant threat to the health of the population and the national economy. Therefore, measures are needed to address the higher incidence rates of hepatitis A among vulnerable populations, and we recommend that governments in developing countries focus on improving sanitation (e.g., creating a purified water supply) and implementing national immunization programs to address future epidemiological changes in hepatitis A.

## Figures and Tables

**Table 1.** Distribution of hepatitis A incidence according to year and sex in Korea, 2002-2012

		Year											Overall
		2002	2003	2004	2005	2006	2007	2008	2009	2010	2011	2012	
Sex	Male	4,775	5,190	5,521	7,278	10,218	9,736	22,279	47,160	37,138	26,180	17,376	192,851
	Female	3,758	4,299	4,725	5,851	7,742	7,623	17,034	36,254	31,465	22,463	15,917	157,131
Age	0-9	1,258	1,132	1,035	1,034	1,163	1,096	1,408	1,832	1,558	1,272	1,095	13,883
	10-19	961	948	1,070	1,334	1,918	1,613	3,404	7,412	6,767	4,914	3,362	33,703
	20-29	1,874	2,162	2,380	3,474	5,440	4,757	12,092	24,885	17,476	11,830	6,805	93,175
	30-39	1,675	1,805	2,150	2,896	4,601	4,586	12,791	28,206	21,014	14,194	8,147	102,065
	40-49	1,208	1,498	1,519	1,898	2,234	2,351	5,051	11,599	11,203	8,236	6,118	52,915
	50-59	824	1,052	1,034	1,242	1,394	1,509	2,481	5,507	6,486	4,971	4,683	31,183
	60-69	520	630	748	824	760	863	1,308	2,591	2,696	2,024	1,945	14,909
	70-79	176	213	246	358	354	458	620	1,112	1,123	935	900	6,495
	80-89	36	46	58	67	89	117	145	239	258	244	216	1,515
	90+	1	3	6	2	7	9	13	31	22	23	22	139
Overall		8,533	9,489	10,246	13,129	17,960	17,359	39,313	83,414	68,603	48,643	33,293	349,982

**Table 2.** Age-standardized hepatitis A incidence rates per 100,000 population according to birth year and sex in Korea, 2002-2012

Sex	Birth year	Year										
		2002	2003	2004	2005	2006	2007	2008	2009	2010	2011	2012
Overall	≤1920	4.89	5.54	7.29	8.42	11.35	11.95	13.27	26.56	18.19	14.37	11.76
	1921-1923	11.47	11.92	11.06	8.61	13.48	19.11	25.37	34.12	25.81	24.93	17.49
	1924-1926	9.24	7.10	12.38	14.37	15.48	16.56	17.70	22.40	22.26	28.23	19.35
	1927-1929	9.68	12.89	12.05	16.25	13.06	17.22	19.77	38.09	34.45	24.52	19.03
	1930-1932	12.48	15.23	12.06	18.67	17.91	22.55	22.97	36.49	31.92	28.72	27.88
	1933-1935	14.05	13.99	16.93	20.31	16.62	19.32	29.55	43.50	42.74	29.93	24.25
	1936-1938	13.13	20.74	20.29	20.26	19.71	21.95	28.58	47.42	41.31	31.16	30.48
	1939-1941	15.65	14.54	18.93	22.24	18.62	21.60	30.23	52.93	52.43	40.73	31.37
	1942-1944	18.30	20.36	23.46	23.08	22.18	24.44	33.88	65.66	57.44	44.18	38.75
	1945-1947	18.75	22.25	19.90	22.96	23.60	22.41	31.03	60.70	57.90	48.00	44.30
	1948-1950	17.73	23.20	23.41	25.30	26.69	26.32	38.96	75.48	81.05	48.58	47.15
	1951-1953	15.14	23.23	20.22	22.50	22.23	25.30	41.68	80.34	75.54	52.41	46.58
	1954-1956	16.08	17.95	17.87	22.77	26.41	25.01	39.95	85.81	95.93	68.60	56.55
	1957-1959	15.39	19.02	19.17	21.94	22.81	25.45	42.29	96.21	94.89	66.42	59.32
	1960-1962	16.12	18.82	19.62	23.64	26.17	27.34	49.35	108.77	113.61	76.03	68.29
	1963-1965	15.81	17.00	18.32	23.52	29.06	27.75	54.43	117.39	106.96	80.86	65.21
	1966-1968	18.42	17.72	18.98	23.87	32.11	31.95	78.23	152.16	127.03	88.09	66.56
	1969-1971	21.44	22.43	24.02	31.20	47.12	43.11	111.23	241.20	180.70	117.24	71.77
	1972-1974	25.43	26.38	30.36	40.04	61.83	59.19	152.66	313.71	225.61	152.47	90.26
	1975-1977	25.54	29.93	32.63	46.07	71.71	65.77	188.02	400.29	264.01	163.27	87.06
	1978-1980	22.19	28.65	34.92	50.21	82.89	72.45	212.13	459.99	301.59	195.84	109.41
	1981-1983	18.24	22.38	28.14	50.50	86.24	78.10	199.76	455.76	336.19	226.28	122.11
	1984-1986	15.59	19.26	22.63	32.04	47.70	51.26	154.97	352.28	259.44	193.97	115.20
	1987-1989	12.49	13.98	17.18	21.51	38.51	36.54	79.13	217.82	199.62	153.94	95.91
	1990-1992	13.05	11.22	12.24	18.03	26.40	24.17	62.25	157.12	162.11	126.06	85.45
	1993-1995	11.46	10.32	12.92	15.61	22.83	20.04	46.11	111.39	105.91	91.78	75.43
	1996-1998	11.67	9.48	9.50	12.82	20.58	17.50	34.24	77.12	78.83	62.91	50.71
	1999-2001	23.23	13.92	10.35	9.66	12.34	13.03	25.77	54.19	49.76	44.61	34.22
	2002-2004	67.99	53.64	38.11	22.16	16.26	13.12	18.16	26.95	29.36	29.75	24.12
	2005-2007				59.89	51.69	41.24	30.57	29.09	26.43	20.50	16.12
	2008-2010							60.04	51.80	36.60	23.98	20.82
	≥2011										50.46	42.76
Male	≤1920	5.03	10.64	10.68	14.26	22.53	21.18	9.14	32.72	28.09	7.88	21.51
	1921-1923	21.28	21.56	16.75	13.69	16.69	22.92	38.56	38.86	38.12	31.15	26.54
	1924-1926	14.46	11.72	14.64	24.76	24.11	16.20	29.70	23.87	33.51	25.96	32.82
	1927-1929	9.72	15.12	18.54	22.04	15.87	20.39	19.28	50.61	46.41	32.04	18.89
	1930-1932	17.58	16.48	18.61	24.06	21.86	28.73	26.10	45.16	35.08	29.92	30.83
	1933-1935	18.02	14.44	21.34	26.15	16.39	20.31	33.62	50.09	43.48	29.96	27.83
	1936-1938	16.39	22.93	26.18	21.49	20.27	26.78	29.15	50.53	44.38	35.21	30.06
	1939-1941	19.41	14.67	21.26	24.99	23.23	21.58	33.16	61.15	53.84	47.21	31.23
	1942-1944	20.33	22.92	22.69	24.44	23.66	24.77	34.20	71.69	61.29	43.07	40.91
	1945-1947	19.68	23.78	18.24	22.90	25.33	21.98	29.13	60.82	58.15	48.35	44.75
	1948-1950	17.21	19.62	21.50	26.49	25.88	27.42	35.69	75.48	80.56	45.87	47.63
	1951-1953	15.04	20.80	19.35	22.93	21.98	25.80	39.10	75.64	70.38	53.57	43.38
	1954-1956	17.04	18.28	19.78	23.74	28.32	24.55	37.16	81.23	89.49	62.24	52.39
	1957-1959	16.70	19.68	18.74	24.57	21.90	24.65	43.06	96.58	93.65	63.58	56.82
	1960-1962	16.32	19.40	20.76	25.00	29.50	30.39	52.24	118.61	111.99	73.91	63.48
	1963-1965	18.08	19.36	18.70	26.19	34.44	31.77	61.96	135.28	114.29	81.08	62.59
	1966-1968	21.32	19.99	20.91	28.04	37.90	36.71	92.30	179.43	137.09	97.08	72.06
	1969-1971	23.99	24.60	25.02	35.43	56.19	51.01	132.80	288.72	204.34	131.88	76.20
	1972-1974	28.07	29.59	32.94	43.43	73.56	70.13	184.05	376.18	261.20	171.55	100.27
	1975-1977	27.75	33.03	34.09	51.31	86.45	76.20	224.40	480.91	294.21	186.13	93.51
	1978-1980	22.42	30.55	35.68	52.33	93.92	78.91	238.61	529.46	331.75	224.05	118.68
	1981-1983	18.29	21.66	25.68	50.71	90.03	83.76	221.81	500.62	352.30	238.65	129.27
	1984-1986	18.75	20.92	24.73	33.89	40.37	48.20	158.70	353.92	263.04	196.39	113.27
	1987-1989	14.49	18.23	22.58	24.67	45.67	39.98	75.94	195.24	186.16	140.75	93.44
	1990-1992	16.81	14.62	15.12	22.52	32.48	28.11	69.07	167.78	162.53	123.10	73.03
	1993-1995	13.21	11.88	15.89	18.82	30.32	26.68	54.05	125.05	120.64	101.35	82.24
	1996-1998	11.54	10.51	12.10	13.67	24.05	22.32	38.91	88.85	92.52	71.99	56.14
	1999-2001	26.35	14.82	10.90	10.58	13.45	15.00	28.11	60.12	59.17	52.08	41.88
	2002-2004	79.06	63.07	41.27	25.45	16.76	15.47	19.70	29.94	32.47	32.13	27.18
	2005-2007				73.19	55.88	44.76	32.82	30.40	24.80	19.66	16.60
	2008-2010							69.73	58.66	37.54	24.33	23.44
	≥2011										55.06	45.84
Female	≤1920	4.84	3.63	6.06	6.44	7.59	8.90	14.59	24.67	15.14	16.29	8.88
	1921-1923	6.63	7.47	8.41	6.28	12.07	17.53	20.39	32.32	21.17	22.67	14.37
	1924-1926	6.47	4.65	11.22	9.30	11.54	16.72	12.32	21.76	17.62	29.07	14.38
	1927-1929	9.65	11.62	8.64	13.22	11.57	15.57	20.01	32.50	29.09	21.13	19.10
	1930-1932	9.37	14.44	7.84	15.25	15.55	19.20	21.23	31.50	30.13	28.09	26.50
	1933-1935	10.98	13.66	13.81	16.40	16.79	18.62	26.76	39.25	42.29	29.91	21.95
	1936-1938	10.48	19.02	15.57	19.26	19.28	18.27	28.16	45.11	38.98	28.22	30.78
	1939-1941	12.33	14.42	16.91	19.91	14.83	21.61	27.77	46.22	51.32	35.75	31.49
	1942-1944	16.41	18.03	24.15	21.87	20.83	24.14	33.60	60.53	54.12	45.15	36.91
	1945-1947	17.87	20.76	21.53	23.02	21.97	22.80	32.82	60.59	57.68	47.69	43.90
	1948-1950	18.26	26.77	25.31	24.11	27.48	25.23	42.13	75.48	81.53	51.17	46.68
	1951-1953	15.24	25.70	21.10	22.06	22.47	24.80	44.22	84.99	80.63	51.27	49.71
	1954-1956	15.10	17.61	15.92	21.79	24.48	25.47	42.79	90.42	102.36	74.88	60.70
	1957-1959	14.03	18.33	19.62	19.24	23.74	26.27	41.50	95.84	96.15	69.30	61.85
	1960-1962	15.91	18.22	18.43	22.23	22.71	24.19	46.38	98.56	115.29	78.22	73.19
	1963-1965	13.42	14.53	17.93	20.73	23.34	23.54	46.65	98.87	99.31	80.63	67.96
	1966-1968	15.38	15.37	17.00	19.55	26.10	26.99	63.64	124.01	116.57	78.75	60.83
	1969-1971	18.79	20.17	22.99	26.81	37.81	34.91	88.81	191.80	156.09	102.18	67.16
	1972-1974	22.70	23.03	27.66	36.52	49.64	47.88	119.99	248.52	188.52	132.61	79.89
	1975-1977	23.25	26.69	31.10	40.60	56.35	55.00	150.04	316.61	232.53	139.48	80.40
	1978-1980	21.94	26.66	34.13	47.98	71.33	65.65	184.41	387.48	270.13	166.45	99.70
	1981-1983	18.20	23.15	30.76	50.29	82.20	72.11	176.64	408.43	319.13	213.22	114.56
	1984-1986	12.17	17.46	20.36	30.05	55.57	54.56	150.91	350.52	255.57	191.40	117.25
	1987-1989	10.27	9.27	11.23	17.99	30.54	32.73	82.64	242.54	214.52	168.55	98.64
	1990-1992	8.82	7.38	8.97	12.95	19.59	19.75	54.55	145.00	161.63	129.37	99.35
	1993-1995	9.50	8.56	9.57	11.97	14.33	12.56	37.23	96.00	89.23	80.95	67.78
	1996-1998	11.82	8.32	6.63	11.89	16.74	12.13	29.04	64.18	63.82	52.87	44.66
	1999-2001	19.81	12.93	9.75	8.65	11.12	10.87	23.22	47.71	39.49	36.46	25.85
	2002-2004	55.85	43.32	34.66	18.59	15.72	10.56	16.49	23.69	25.99	27.17	20.81
	2005-2007				45.47	47.17	37.46	28.16	27.68	28.17	21.41	15.60
	2008-2010							49.69	44.49	35.60	23.60	18.04
	≥2011										45.58	39.49

**Table 3.** Model fitness for age-period-cohort analyses of hepatitis A incidence rates according to sex in Korea, 2002-2012

	Model	df	-2LLR	AIC	p-value
Overall	Age	84	189,651	189,707	< 0.001
	Period	108	147,011	147,019	< 0.001
	Cohort	81	189,421	189,483	< 0.001
	Age + period	81	15,684	15,686	< 0.001
	Age + cohort	54	86,853	86,855	< 0.001
	Period + cohort	78	21,404	21,472	< 0.001
	Age + period + cohort (IE)	51	5,977	5,981	Reference
Male	Age	84	105,170	105,226	< 0.001
	Period	108	81,423	81,431	< 0.001
	Cohort	81	104,807	104,869	< 0.001
	Age + period	81	9,458	9,460	< 0.001
	Age + cohort	54	50,182	50,184	< 0.001
	Period + cohort	78	12,183	12,251	< 0.001
	Age + period + cohort (IE)	51	3,463	3,467	Reference
Female	Age	84	85,541	85,597	< 0.001
	Period	108	67,438	67,446	< 0.001
	Cohort	81	86,035	86,097	< 0.001
	Age + period	81	6,368	6,370	< 0.001
	Age + cohort	54	36,657	36,659	< 0.001
	Period + cohort	78	10,673	10,741	< 0.001
	Age + period + cohort (IE)	51	2,513	2,517	Reference

df, degrees of freedom; LLR, likelihood ratio; AIC, Akaike information criterion; IE, intrinsic estimator.
